# 

**Published:** 1924-05

**Authors:** 


					New Series. Vol. III. No. 32 May, 1924. Monthly, Price 6d. C05J^EE]

				

